# Distinctive characteristics of bronchial reticular basement membrane and vessel remodelling in chronic obstructive pulmonary disease (COPD) and in asthma: they are not the same disease

**DOI:** 10.1111/j.1365-2559.2011.04147.x

**Published:** 2012-05

**Authors:** Amir Soltani, Hans Konrad Muller, Sukhwinder S Sohal, David W Reid, Steve Weston, Richard Wood-Baker, Eugene Haydn Walters

**Affiliations:** 1 Respiratory Research Group, University of TasmaniaHobart; 2 Descipline of Pathology, Menzies Research Institute, University of TasmaniaHobart; 3 Queensland Institute of Medical ResearchBrisbane; 4 Mercy Healthcare VictoriaVictoria, Australia

**Keywords:** airway remodelling, asthma, bronchial biopsies, chronic obstructive pulmonary disease, reticular basement membrane

## Abstract

**Aims:**

This study compared reticular basement membrane (Rbm) and vascular remodelling within the bronchial mucosa of subjects with chronic obstructive pulmonary disease (COPD) with those from patients with asthma, to test the ‘Dutch hypothesis’ of whether these are essentially the same or different pathological conditions.

**Methods and results:**

Bronchoscopic biopsies were stained with anti-collagen IV antibody; 18 current smoking COPD, 10 symptomatic asthmatics and 13 healthy non-smoking controls were studied. The Rbm in COPD was fragmented, non-homogeneous, variable in thickness and hypervascular, whereas in asthma the Rbm was compact and homogeneous with no evidence of increased vascularity compared to controls. Length of Rbm splitting presented as percentage of Rbm length was used to measure fragmentation; it was greater in COPD than in controls and asthmatics [median (range) 20.7% (0.4–68.5) versus 5.3% (0.0–21.7) versus 1.5% (0.0–15.1), *P* < 0.001]. The number of Rbm vessels/mm Rbm [median (range) 10.1 (1.6–23.0) versus 4.5 (0.0–26.4) versus 4.4 (0.4–8.1), *P* < 0.01] and area of Rbm vessels, μm^2^/mm Rbm [median (range) 953 (115–2456) versus 462 (0–3263) versus 426 (32–2216), *P* < 0.05] was also increased in COPD compared to normal subjects and asthmatics.

**Conclusions:**

The characteristics of Rbm remodelling are quite different in asthma and COPD.

## Introduction

Asthma and chronic obstructive pulmonary disease (COPD) are common chronic inflammatory airway diseases.^1^,^2^ They are both characterized by airflow limitation and structural changes in the airway components, known as remodelling,^3^,^4^ although this has been relatively little studied in COPD. Although they differ in many aspects, it has been suggested repeatedly that they share common pathological features (the ‘Dutch hypothesis’).^5^ Asthma is mainly immunologically mediated and characterized by bronchial hyper-responsiveness and reversible airway obstruction, while COPD is caused by exposure to cigarette smoking or other noxious gases and generally follows an unremitting progressive course.^6^,^7^ Examination of bronchial biopsies (BB) in these two diseases has revealed different inflammatory cell profiles and structural changes.^8^–^15^

Over several decades, investigators have used mainly surgically derived tissues to investigate changes in the airways of asthma and COPD patients. These studies have shown epithelial shedding, increased reticular basement membrane (Rbm) thickness, inflammatory cell infiltration, increased lamina propria vessels, smooth muscle layer thickening and mucous gland hyperplasia in asthma. In COPD, the main changes in the airways have been described as epithelial cell metaplasia, goblet cell hyperplasia, inflammatory cell infiltration and submucosal gland hyperplasia.^13^–^20^

In the last two decades, BB have been used increasingly to study bronchial mucosal remodelling in asthma; these have been able to add details about hypervascularity of the lamina propria^21^–^25^ and Rbm thickening^26^–^31^ and their response to therapy.^26^,^32^ However, reports in COPD using BB are much more limited.^1^,^33^–^35^

Reports on large airway mucosal vessels in COPD have not been in agreement, and varied from describing hypovascularity of the lamina propria in current smokers with COPD^36^ to increased vessels in the lamina propria in smokers with chronic bronchitis^37^ or increased vascular area, but normal vessel numbers in ex-smokers with COPD who had quit for longer than 10 years.^38^ We have recently reported that lamina propria vessels in smokers are decreased in number, but vessels associated with the Rbm are increased, especially in those with COPD.^36^

Reports on Rbm thickness in COPD are also conflicting.^30^,^39^ We have described Rbm thickness to be very variable in COPD, but overall not significantly different from non-smoking controls.^36^In asthma, Rbm morphology has been described as homogeneous and hyaline in appearance.^26^,^40^ Our group has recently published a comparison of BB from COPD subjects and healthy controls and has reported that the Rbm is fragmented and hypervascular in COPD.^36^ As far as we are aware, there has been no other study of Rbm morphology and vascularity in COPD, although a 2009 publication indicated changes in Rbm matrix proteins.^30^ In the current study, we have conducted a detailed analysis of pathological changes within the Rbm and associated blood vessels in COPD versus asthma as well as normal control groups, with all procedures and processing being identical. This has not been attempted previously. We did not observe fragmentation of the Rbm or abnormal vascular changes in juxtaposition to the Rbm in our previous work in asthma, but we did not look specifically for these changes. We have therefore undertaken a formal comparison between COPD and asthma to confirm fully that these Rbm changes are specific for COPD, and also to test formally the ‘Dutch hypothesis’ about the similarity of disease processes.

## Materials and methods

This was a cross-sectional between-group comparative study approved by the Human Research Ethics Committee (Tasmania) Network and all subjects provided written informed consent.

### Subjects

Subjects were recruited through advertisement. Eighteen current smokers who had mild to moderate COPD (GOLD I and II), 10 currently symptomatic asthmatics and 13 normal controls were recruited. COPD was diagnosed according to the GOLD guidelines.^7^ COPD subjects did not have a history of asthma or other respiratory diseases and were only on anti-cholinergic bronchodilator medications. All asthmatics met the American Thoracic Society (ATS)^41^ criteria for asthma and had measurable hyper-responsiveness with methacholine (PD_20_ < 2 mg). Asthmatics were on low-dose inhaled corticosteroids (equivalent to 200–300 μg of beclomethasone dipropionate daily) but still had symptomatic active disease. Controls were non-smokers and did not have any history or other manifestation of a respiratory disease and had normal spirometry. All subjects underwent fibreoptic bronchoscopy with biopsies.

### Lung Function Tests

Lung function tests were performed according to the ATS/ERS guidelines.^42^

### Fibreoptic Bronchoscopies and Biopsies

Fibreoptic bronchoscopies and biopsies were performed as described previously.^25^ There were no complications from the procedures.

### Tissue Processing

Four biopsies were fixed in 4% neutral buffered formalin for 2 h and subsequently processed into paraffin through graded alcohol and xylene using a Leica ASP 200 tissue processor (Leica Microsystems GmbH, Wetzlar, Hesse, Germany). Sections were cut at 3 μ from individual paraffin blocks, stained with haematoxylin and eosin and assessed morphologically for immunostaining. Blocks stained were chosen to minimize tangential sectioning of the epithelium and to provide greatest length of epithelium for assessment. Two 3-μ sections from appropriate blocks were collected on each slide being separated by a minimum of 50 μ. Following removal of paraffin and hydration to water, immunostaining for collagen-IV (cat. no. M0785 clone CIV 22: 1/100 dilution, 90 min at room temperature with heat retrieval; Dako Cytomation, Copenhagen, Denmark) was performed on separate slides. In each case a non-immune immunoglobulin (Ig)G1-negative control (X0931 clone DAKGO1; Dako Cytomation) was performed to eliminate false positive staining. Bound antibodies were elaborated using peroxidase-labelled Envision + (cat. no. K4001; Dako Cytomation) and liquid diamonobenzidene (DAB) + (cat. no. K3468; Dako Cytomation).

### Measurements

Measurements were performed by using a computer-assisted image analysis tool (Image-Pro version 5.1; Media Cybernetics, Bethesda, MD, USA). Pictures of all intact and non-overlapping areas were taken from each slide and eight separate fields were chosen randomly for assessment and endpoint quantitation. The person who performed the assessments and analyses (A.S.) was blinded to the diagnoses and slides order.

**Figure 1:**
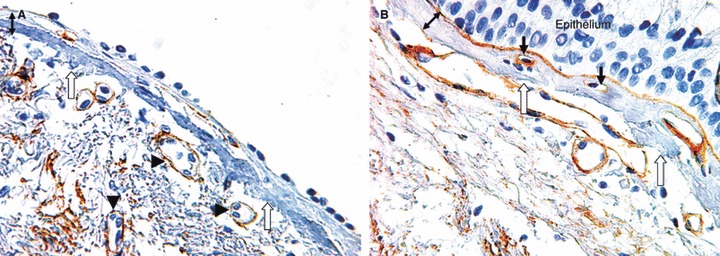
Reticular basement membrane (Rbm) splitting and vessels. **A** and **B** represent micrographs from bronchial biopsies in two COPD patients. The Rbm (two-headed arrows) is attached below the true basement membrane; in COPD it is fragmented and shows splitting (white arrows). Black arrows indicate Rbm-associated vessels. Arrow heads indicate vessels in the lamina propria. Bronchial biopsies, anti-Collagen IV antibody staining.

Fragmentation of the Rbm was associated with splitting and formation of clefts.^36^ Quantitation of this splitting was used as an index of fragmentation (Figure 1). Thus, the total length of splits was measured and normalized to the length of Rbm.

**Figure 2:**
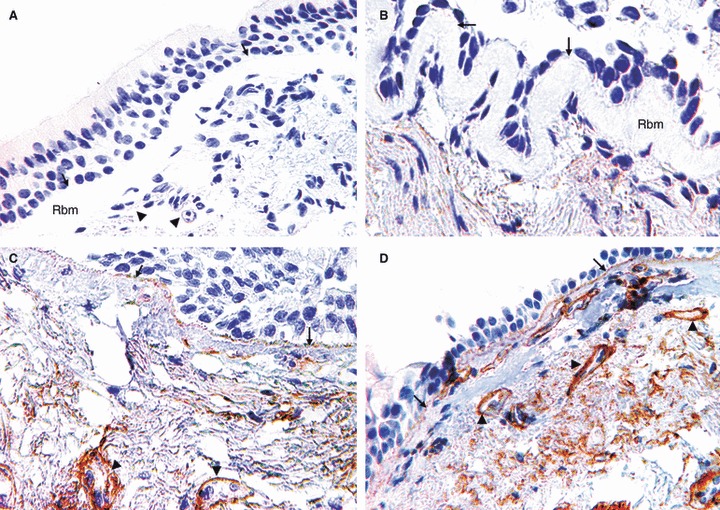
Morphology of the reticular basement membrane (Rbm) in asthma (**A** & **B**) compared to COPD (**C** & **D**). The Rbm, as indicated in **A** & **B** is located immediately beneath the true epithelial basement membrane (black arrows). **A** & **B** represent micrographs from airway biopsies in two asthma subjects. The Rbm is compact, homogenous and thickened in asthma. **C** & **D** represent micrographs from airway biopsies in two COPD subjects. In COPD the Rbm is non-homogenous, variable in thickness and fragmented. Vessels are seen contacting and partially or completely embedded within the Rbm in **D**. Vessels in the lamina propria are indicated with arrowheads. Bronchial biopsies, anti-Collagen IV antibody staining.

Vessels in the Rbm included those in contact with its anti-lumenal surface, those partly surrounded by and those totally embedded within the Rbm (Figures 1 and 2). The number and cross-sectional area of these vessels, stained with anti-collagen IV antibody, were measured. The cross-sectional area of vessels was assessed as the area enclosed by the anti-collagen IV antibody staining of the endothelial basement membrane. All measurements were divided by the length of Rbm. Slides have also been stained for Von Willebrand factor and a comparison made of vessels quantified both ways; comparability was excellent (data not shown).

### Statistical Analyses

Non-parametric analysis of variance (anova) (Kruskal–Wallis) and *post-hoc* Mann–Whitney *U*-tests were used for testing mean differences between groups. All analyses were performed by pasw statistics 18. Two-tailed *P*-values<0.05 were considered significant.

## Results

Table 1 summarizes participants’ demographics. COPD subjects were significantly older than the other groups. Ten subjects with COPD suffered from mild disease [GOLD I, forced expiratory volume in 1 s (FEV_1_)/forced vital capacity (FVC)<70% but FEV_1_ > 80% predicted] and eight had moderate airflow limitation (GOLD II, FEV_1_/FVC<70% and FEV_1_ 50–79% predicted). Gender distribution was not different between groups. Mean pack-year smoking history in the COPD group was 51. Mean values for bronchodilator responsiveness [BDR, calculated as (post-bronchodilator FEV_1_ − pre-bronchodilator FEV_1_) divided by pre-bronchodilator FEV_1_ × 100] were 8% and 18% for the COPD and asthmatic subjects, respectively. All COPD subjects were current smokers. Four of the asthmatics had never smoked and six were ex-smokers who had quit for longer than 1 year. Controls had never smoked.

**Table 1:** Demographics

Groups (numbers)	H-N (13)	S-COPD (18)	Asthma (10)
Age, median (range)	54 (20–68)	61 (46–78)	40 (27–58)

Gender, female%	62	39	30

FEV1/FVC ratio%, median (range)	82 (71–88)	58 (46–68)	70 (58–80)

COPD, Chronic obstructive pulmonary disease; FEV1, forced expiratory volume in 1 s; FVC, forced vital capacity.

**Figure 3:**
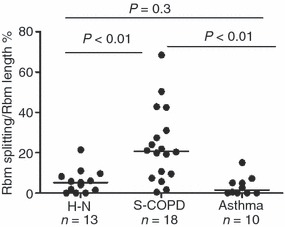
Length of splitting compared between groups. H-N, healthy nonsmokers; S-COPD, Smokers with COPD; Rbm, reticular basement membrane.

The morphology of the Rbm was different in COPD compared to asthma and both were, in turn, different from normal. In COPD, the Rbm was non-homogeneous and fragmented, but in asthma it was homogeneous and compact (Figures 1 and 2). The length of Rbm splits/length of the Rbm expressed as a percentage and presented as median (range) was greater in the COPD group [20.7% (0.4–68.5)] compared to both the control [5.3% (0.0–21.4)] and asthma [1.5% (0–15.1)] groups (Figure 3).

**Figure 4:**
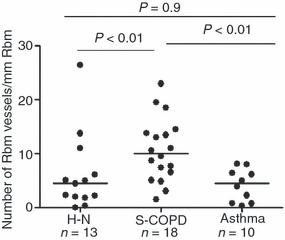
Number of vessels compared between groups. H-N, healthy nonsmokers; S-COPD, Smokers with COPD; Rbm, reticular basement membrane.

**Figure 5:**
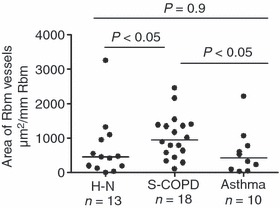
Cross-sectional area of vessels compared between groups. H-N, healthy nonsmokers; S-COPD, Smokers with COPD; Rbm, reticular basement membrane.

There were more Rbm-associated vessels, expressed as number of vessels per mm length of the Rbm and presented as median (range) in the COPD group [10.1 (1.6–23.0)], compared to the control [4.5 (0.0–26.4)] and asthma [4.4 (0.4–8.1)] groups. The cross-sectional area of vessels, expressed as μm^2^/mm length of the Rbm and presented as median (range) in the COPD group [953 (115–2456)] was approximately twice that observed in the control [462 (0–3263)] and asthma [426 (32–2216)] groups (Figures 4 and 5).

## Discussion

The core part of this study compared Rbm remodelling and related vascular changes in COPD and asthma and has confirmed that the Rbm is fragmented and hypervascular in COPD, but distinctly different in asthma. In contrast to COPD, in asthma the Rbm is compact and homogeneous. We have shown previously that the Rbm is thicker in asthma, at least before using inhaled corticosteroid.^26^ The ‘Dutch hypothesis’, which suggests essentially that asthma and COPD are pathologically the same disease presenting in different ways, appears to us incorrect. We believe that we have demonstrated that the changes we have described in COPD^36^ are real and specific, and not confounded by some sort of processing artefact.

Only a few studies have used BB to examine airway remodelling in COPD, and these focused mainly on lamina propria vessels or thickness of the Rbm.^37^,^39^ As far as we know, the current study is the first that has directly compared and contrasted the morphology of the Rbm and its vessel content between COPD, asthma and normal controls using bronchial biopsies, which experience has shown can be highly informative.

We have suggested that Rbm fragmentation in current smokers with COPD is the result of the action of matrix metalloproteinases and may be a manifestation of epithelial–mesenchymal transition (EMT),^43^ with transitioning epithelial cells digesting their way through to the lamina propria. Rbm fragmentation correlated with cigarette smoking, which suggested that smoking has the potential to induce such changes in COPD airways.^36^ We have not stained the asthmatic sections in this study for matrix metalloproteinase, because there are very few cells in the Rbm in asthma to comment on.

Increased vascularity of the lamina propria has been reported in asthma and its association with angiogenic factors has been described.^25^ However, this current study showed that this hypervascularity of deeper structures is not associated with an increase in Rbm vessels. Such data on COPD using BB are very limited. Calabrese *et al.*^37^ found an increased vessel number and more vascular endothelial growth factor (VEGF)-positive cells in the lamina propria of smokers with COPD and chronic bronchitis compared to normal controls. Zanini *et al.*^38^ compared BB from ex-smokers with COPD who had quit for longer than 10 years with controls and did not find any difference in the number of vessels, but the vascular area was larger and VEGF-positive cells, transforming growth factor-positive (TGF-β) cells and fibroblast growth factor (FGF)-positive cells were increased in COPD. We have reported lower vessel numbers in the lamina propria but hypervascularity of the Rbm in current smokers with COPD. Vessel-associated VEGF and TGF-β activity were also increased in the Rbm of COPD subjects. Very unlike asthma, we suggested that angiogenic activity is augmented in the Rbm, but reduced in the lamina propria, and proposed that more studies are needed to assess other angiogenic factor activities in COPD and smokers.^36^

We cannot explain all the differences between these various, although limited, studies in COPD. However, different and in some cases contradictory results from investigations may reflect the diversity of presentations of COPD; for example, the severity of airflow limitation, presence of chronic bronchitis and emphysema and the smoking status of participants. Our COPD patients were also older than those in the other groups. This may be a confounding factor, but we did not find any correlations between our findings and age in this group with quite a wide age range.

In summary, our study shows that Rbm remodelling is different in COPD and asthma; pathologically, they are quite distinct. The Rbm has been defined clearly using BB and is homogeneous and compact in asthma but non-homogeneous, fragmented and hypervascular in COPD. The structural components of the Rbm in these two diseases need to be evaluated further and related to physiological abnormalities. The pathological picture in COPD is highly suggestive of active EMT.

## Abbreviations

ATS: American Thoracic Society
BB: bronchoscopic biopsies
COPD: chronic obstructive pulmonary disease
EMT: epithelial–mesenchymal transition
ERS: European Respiratory Society
FEV_1_: forced expiratory volume in 1 s
FGF: fibroblast growth factor
FVC: forced vital capacity
GOLD: global initiative for chronic obstructive lung disease
Rbm: reticular basement membrane
TGF-β: transforming growth factor beta
VEGF: vascular endothelial growth factor
